# Sleep psychoeducation strategies for patients with chronic migraine and sleep quality complaints

**DOI:** 10.1055/s-0046-1817029

**Published:** 2026-03-11

**Authors:** Daniel de Godoy Andreis, Maria Lúcia Ferreira Rodrigues, Alessandra Zanatta, Ivo José Monteiro Marchioro, Pedro André Kowacs

**Affiliations:** 1 Universidade Federal do Paraná, Hospital das Clínicas, Curitiba PR, Brazil.; 2 Instituto de Neurologia de Curitiba, Serviço de Neurologia, Curitiba PR, Brazil.

**Keywords:** Migraine Disorders, Sleep Initiation and Maintenance Disorders, Sleep Hygiene

## Abstract

**Background:**

Chronic migraine is a prevalent and debilitating condition, frequently associated with sleep disorders, particularly insomnia.

**Objective:**

To evaluate the impact of sleep psychoeducation measures on clinical aspects of chronic migraine patients with sleep quality complaints, under the hypothesis that such interventions would benefit both migraine symptoms and insomnia difficulties.

**Methods:**

A total of 100 patients from our neurology service's headache outpatient clinic were screened using an author-developed questionnaire addressing sleep quality complaints. The intervention group included 68 patients with sleep disturbance complaints, who completed the Pittsburgh Sleep Quality Index (PSQI), the Epworth Sleepiness Scale (ESS), and the Insomnia Severity Index (ISI), and subsequently received psychoeducation guidelines and individualized recommendations. The control group consisted of 32 patients without sleep disorder complaints, from whom clinical data on migraine were collected. After 2 to 3 months, the questionnaires were reapplied to the intervention group, and medical records from both groups were reviewed for comparison.

**Results:**

In the control group, there was no statistically significant change in headache frequency or intensity. In the intervention group, a significant reduction was observed in both parameters. Regarding sleep disturbance, the intervention group showed a significant reduction in PSQI (from 13.7 to 11.7) and ISI (from 18.5 to 13.4), but no significant variation in ESS.

**Conclusion:**

Sleep psychoeducation, supported by an informative booklet, produced positive outcomes not only in sleep parameters but also in reducing the frequency and intensity of headaches in chronic migraine sufferers.

## INTRODUCTION

Among primary headaches, migraine is one of the most prevalent, affecting approximately 15% of the global population. 1 2 The prevalence of headache disorder is of more than 50%, with 14% for migraine, and 30% for tension-type headache. 2 Migraine prevalence estimates showed an upward trend over the years, whereas estimates of all headache types varied around the world. 2 It also impairs concentration and productivity while leading to chronic pain and irritability. 1 3

A significant complication of migraine is its chronification, defined as headaches occurring on 15 or more days per month for more than 3 months, with at least 8 days per month of headaches with migraine features. 4 This transition is attributed to maladaptive changes in central pain modulation within the brainstem and encephalon, leading to worsening symptoms and increased disability. 1 5

There is a well-documented bidirectional relationship between headache and sleep disturbances, with some headaches occurring exclusively in relation to sleep and vice versa. 6 Chronic migraine, in particular, has been closely associated with sleep disorders, although the underlying mechanisms remain incompletely understood.

Insomnia, defined as a persistent difficulty in initiating or maintaining sleep accompanied by daytime impairment (fatigue, mood changes, cognitive dysfunction, and reduced quality of life), 7 is one of the most prevalent sleep disorders and is closely associated with chronic migraine. Around 1/3 adults report insomnia symptoms, and approximately 12% meet the diagnostic criteria for chronic insomnia. 8 9

Individuals with migraine show a significantly higher prevalence of insomnia compared with those without, 10 underscoring the bidirectional relationship between these conditions. Sleep disturbances may promote migraine chronification by increasing cortical hyperexcitability, pain sensibility, and dysfunction of descending inhibitory pain pathways. 7 11 12 Conversely, recurrent migraine attacks, pain anticipation, and the resulting anxiety or hyperarousal can interfere with sleep onset and maintenance, exacerbating both disorders. 13 14 15

Nonpharmacological interventions for sleep disorders, particularly insomnia, have demonstrated significant benefits. 6 7 8 Pharmacological treatments are generally recommended only for short-term use due to risks of tolerance and dependence. 16 17 18 Cognitive Behavioral Therapy for Insomnia (CBT-I) is the first-line treatment endorsed by the American Academy of Sleep Medicine, emphasizing the importance of behavioral modifications. 18 19 20 This treatment requires trained therapists, involving structured cognitive and behavioral techniques to improve sleep. 18 Alternatively, sleep hygiene—a component of sleep psychoeducation—comprises general behavioral strategies provided by healthcare professionals. 19

Sleep psychoeducation integrates CBT-I principles such as screen time restriction, stimulus control, bedtime regulation and relaxation techniques, but does not require specialized therapists. Unlike CBT-I, which involves multiple consultations, psychoeducation is typically delivered in a single session. 21 22 23

Despite increasing interest in the association between sleep disturbances and chronic migraine, evidence remains limited, and further studies are needed to clarify this relationship. In this context, the intervention selected in the present work was sleep psychoeducation, as it can be delivered within the limited time available in outpatient consultations, whereas CBT-I requires a previously trained professional and significantly more time to administer.

Given the substantial burden of chronic migraine and its probable association with insomnia, this study aims to evaluate the impact of sleep psychoeducation on migraine symptoms among patients at the Neurology Service's Headache Outpatient Clinic, Complexo do Hospital de Clínicas, Universidade Federal do Paraná (CHC-UFPR). Secondary objectives include enhancing understanding of the migraine-insomnia relationship, providing scientific support for nonpharmacological sleep interventions, and assessing the feasibility of implementing sleep psychoeducation in outpatient settings by nonspecialist physicians.

The main hypothesis of this study was that sleep psychoeducation is an effective intervention for patients with sleep quality complaints during outpatient consultations, in addition to the evidence of a probable causal relationship between insomnia and chronic migraine. Finally, we believe that improving sleep quality through psychoeducational measures would positively influence the clinical aspects of chronic migraine.

## METHODS

This study was approved by the Research Ethics Committee, under the CAAE 82292724.6.0000.0096. A nonrandomized interventional study was conducted among patients from the headache outpatient clinic of the Neurology Service at CHC-UFPR, who presented treatment-resistant migraine, defined as chronic migraines that failed three different classes of adequately administered prophylactic treatments. 24

Participants were screened based on sleep quality complaints using an 11-item standardized questionnaire assessing chronic migraine intensity, sleep quality, and prevalent sleep disorder characteristics ( **Supplementary Material I** ). Adults (age ≥ 18 years) who provided a signed informed consent form were included. Patients with chronic migraine and sleep quality (initiation or maintenance) complaints compatible with insomnia formed the intervention group, while those without sleep complaints served as controls. Those with symptoms indicative of sleep apnea were excluded.

### Assessment tools and intervention

For intervention group participants, three validated questionnaires were administered: the Pittsburgh Sleep Quality Index (PSQI), a 25-item tool to assess sleep patterns and disturbances over the past 30 days; the Epworth Sleepiness Scale (ESS), to evaluate excessive daytime sleepiness; and the Insomnia Severity Index (ISI), to measure the severity of insomnia symptoms and associated daytime impairment.

Following detailed anamnesis, including medical and lifestyle factors, participants from the intervention group received individualized sleep psychoeducation from two medical students, who were trained and supervised by their academic supervisor, a sleep medicine doctor, comprising an informational leaflet developed by the research group and personalized recommendations. These recommendations were tailored according to each patient's routine and encompassed sleep hygiene measures such as: limiting screen time, reducing caffeine intake and having light meals before bedtime, engaging in regular aerobic exercise, managing worries, practicing breathing relaxation techniques, establishing a consistent sleep routine and restricting time spent in bed. This process lasted 20 to 30 minutes.

### Follow-up and data analysis

After 2 to 3 months, the intervention group completed follow-up questionnaires assessing migraine intensity and frequency, as well as adherence to sleep psychoeducation, stating which were the most difficult measures of sleep psychoeducation to practice and whether they felt a subjective improvement on migraine symptoms and sleep quality after the intervention. Clinical variables (migraine characteristics, intensity, frequency, and pharmacological treatment changes) were analyzed pre- and postintervention within each group.

The sample size calculation was based on data from the first 10 participants in each group (control and intervention), to detect a significant difference between the groups in terms of reduction in pain intensity. Standard deviations (SD) were estimated at 1.1 in the control group and 2.2 in the intervention group. A minimum difference of one point in reducing pain intensity between groups was considered clinically relevant. Admitting a significance level of 5% (two-tailed), power of 80% and allocation ratio of 2:1 (intervention:control), a sample size of 62 participants in the intervention and 31 in the control group was estimated. Planning to use a nonparametric approach to compare groups, sample sizes were increased by 5%, resulting in 65 participants in the intervention and 33 in the control group.

Data were recorded in Microsoft Excel 2015 (Microsoft Corp.) and analyzed using the IBM SPSS Statistics for Windows (IBM Corp.), version 19.0. Normality of distribution was assessed via Shapiro-Wilk and Kolmogorov-Smirnov tests. Descriptive statistics included mean ± SD for normally distributed variables and median with interquartile range (IQR) for non-normal data. Qualitative variables were expressed as absolute frequencies and percentages.

Comparative analyses were conducted using Fisher's exact test for qualitative variables, Student's t-test for normally distributed continuous variables, and Mann-Whitney's test for non-normally distributed continuous variables. A significance level of *p* < 0.05 was applied.

Data obtained from the questionnaires were used exclusively for this study and will be discarded upon completion.

## RESULTS

This study was carried out with 100 subjects who had chronic migraine, all of whom were treated at the headache outpatient clinic of the Neurology Service of CHC-UFPR, during the second semester of 2024 and the first trimester of 2025. Of these, 32 (32%) had no complaints about sleep quality and were included in the control group, while 68 patients (68%) had sleep complaints and received guidance on sleep psychoeducation, who were designated as the intervention group.

### Group comparison regarding demographic and clinical variables

#### Demographic variables

Among the patients included in the study, 88 were female, 27 belonging to the control group (84.4%) and 61, to the intervention group (89.7%), while 12 were male, 5 allocated to the control group (15.6%) and 7, to the intervention group (10.3%). The mean age of the study patients was 54.5 ± 15.3 years for the control group, and 51.8 ± 13.7 years for the intervention group. Demographic variables did not show statistically significant variation between the control and intervention groups ( *p* = 0.365 for age and 0.514 for sex).

#### Clinical variables

Data on clinical variables were grouped in Table 1 . There was a statistically significant variation between the intervention and control groups in the intensity of headache before the intervention. Also, there was a statistically significant difference in the percentage of patients using topiramate after the intervention, with a greater predominance in the intervention group, which remained relatively constant in relation to the percentage used before the intervention in the same group (29.4 before and 30.9% after).

**Table 1:** Comparison of headache clinical variables between the intervention and control groups, pre- and postintervention

Variable	Classification	Group				*p* -value*
		Control (n = 32)		Intervention (n = 68)		
				
Intensity						
Pre**	[median (minimum–maximum)]	8 (3–10)		9 (4–10)		0.013
Post***	[median (minimum–maximum)]	8 (3–10)		8 (1–10)		0.677
Intensity reduction	(Post–Pre)	0 (−3–3)		1 (−3–−9)		0.009
Frequency						
Pre	Daily	21 (65.6%)		39 (57.4%)		0.073
	4–6x week	2 (6.3%)		4 (5.9%)		
	1–3x week	4 (12.5%)		22 (32.4%)		
	< 1x week	5 (15.6%)		3 (4.4%)		
Post	Daily	17 (53.1%)		14 (20.6%)		–
	4–6x week	3 (9.4%)		0 (0.0%)		
	1–3x week	7 (21.9%)		44 (64.7%)		
	< 1x week	5 (15.6%)		10 (14.7%)		
Change treatment	Change of conduct	23 (71.9%)		42 (61.8%)		0.535
	Treatment maintenance	8 (25.0%)		21 (30.9%)		
	Dose change	1 (3.1%)		5 (7.4%)		
Topiramate						
Pre	No	27 (84.4%)		48 (70.6%)		0.215
	Yes	5 (15.6%)		20 (29.4%)		
Post	No	29 (90.6%)		47 (69.1%)		0.023
	Yes	3 (9.4%)		21 (30.9%)		
Antidepressant						
Pre	No	22 (68.8%)		41 (60.3%)		0.507
	Yes	10 (31.3%)		27 (39.7%)		
Post	No	23 (71.9%)		37 (54.4%)		0.126
	Yes	9 (28.1%)		31 (45.6%)		
Anesthetic block 1						
Pre	No	26 (81.3%)		46 (67.6%)		0.232
	Yes	6 (18.8%)		22 (32.4%)		
Post	No	20 (62.5%)		40 (58.8%)		0.828
	Yes	12 (37.5%)		28 (41.2%)		
Botulinum toxin 2						
Pre	No	27 (84.4%)		57 (83.8%)		1
	Yes	5 (15.6%)		11 (16.2%)		
Post	No	28 (87.5%)		63 (92.6%)		0.462
	Yes	4 (12.5%)		5 (7.4%)		

Notes: *Nonparametric Mann-Whitney test (quantitative variables); Fisher's exact test or chi-squared test (categorical variables); *p* < 0.05; **Pre: Preintervention results; ***Post: Postintervention results; ^1^ Anesthetic block with bupivacaine or lidocaine; ^2^ Application of botulinum toxin in trigger points associated with headache.

In the control group, there was only a statistically significant variation between the data from the initial visit and the follow-up after 2 to 3 months in patients who were indicated for anesthetic blockade to control pain crises ( *p* = 0.034). The other clinical data, both those related to headache frequency and intensity, and those related to the percentage of each different type of treatment, did not show a statistically significant variation before and after the intervention period, as shown in Table 2 .

**Table 2:** Comparison between pre- and postintervention headache clinical variables - Control group

Variable	Classification	Control group (n = 32)				*p* -value*
		Preintervention		Postintervention		
			
Intensity	Median (minimum–maximum)	8 (3–10)		8 (3–10)		0.918
Frequency	Daily	21 (65.6%)		17 (53.1%)		0.262
	4–6x week	2 (6.3%)		3 (9.4%)		
	1–3x week	4 (12.5%)		7 (21.9%)		
	< 1x week	5 (15.6%)		5 (15.6%)		
Topiramate	No	27 (84.4%)		29 (90.6%)		0.500
	Yes	5 (15.6%)		3 (9.4%)		
Antidepressant	No	22 (68.8%)		23 (71.9%)		0.388
	Yes	10 (31.3%)		9 (28.1%)		
Anesthetic block 1	No	26 (81.3%)		20 (62.5%)		0.034
	Yes	6 (18.8%)		12 (37.5%)		
Botulinum toxin 2	No	27 (84.4%)		28 (87.5%)		1
	Yes	5 (15.6%)		4 (12.5%)		

Notes: *Non-parametric Wilcoxon test (intensity); binomial test (dichotomous categorical variables); Stuart-Maxwell symmetry test (categorical variables with more than 2 classifications); *p* < 0.05; ^1^ Anesthetic block with bupivacaine or lidocaine; ^2^ Onabotulinum A toxin in trigger points of headache.

In the intervention group, a statistically significant variation can be observed in the following clinical data: reduction of headache intensity; reduction of headache frequency; and percentage of patients who underwent botulinum toxin application, with a reduction of 8.8% in relation to the preintervention value. The other clinical data did not demonstrate a statistically significant change between the pre- and postintervention periods ( Table 3 ).

**Table 3:** Comparison between pre- and postintervention clinical variables in the intervention group

Variable	Classification	Intervention group (n = 68)				*p* -value*
		Preintervention		Postintervention		
			
Intensity	Median (minimum–maximum)	9 (4–10)		8 (1–10)		< 0.001
Frequency	Daily		39 (57.4%)		14 (20.6%)	< 0.001
	4-6x week		4 (5.9%)		0 (0.0%)	
	1-3x week		22 (32.4%)		44 (64.7%)	
	< 1x week		3 (4.4%)		10 (14.7%)	
Topiramate	No		48 (70.6%)		47 (69.1%)	1
	Yes		20 (29.4%)		21 (30.9%)	
Antidepressant	No		41 (60.3%)		37 (54.4%)	0.125
	Yes		27 (39.7%)		31 (45.6%)	
Anesthetic block	No		46 (67.6%)		40 (58.8%)	0.234
	Yes		22 (32.4%)		28 (41.2%)	
Botulinum toxin	No		57 (83.8%)		63 (92.6%)	0.031
	Yes		11 (16.2%)		5 (7.4%)	

Notes: *Nonparametric Wilcoxon test (intensity); binomial test (dichotomous categorical variables); Stuart-Maxwell symmetry test (categorical variables with more than 2 classifications); *p* < 0.05.

Additionally, it is worth noting that, in a subjective evaluation conducted through open-ended questions in the follow-up form applied 2 to 3 months after the intervention, 61.8% of patients reported improvement in sleep quality and 58.8% reported improvement in headaches when specifically asked about the impact of sleep psychoeducation on their daily routine.

### Group comparison regarding the clinical characteristics of chronic migraine, before and after the intervention

#### Pain frequency

The clinical data associated with the frequency of pain before and after the intervention in the control and intervention groups were detailed. The tables show, for each group, the frequencies and percentages of cases according to the classifications of the evolution from pre to post intervention, considering the following frequencies of headache: “Daily”, “4 to 6 times a week”, “1 to 3 times a week” and “< 1 time a week”. These data are presented in Tables 2 and 3 .

**Figure 1:**
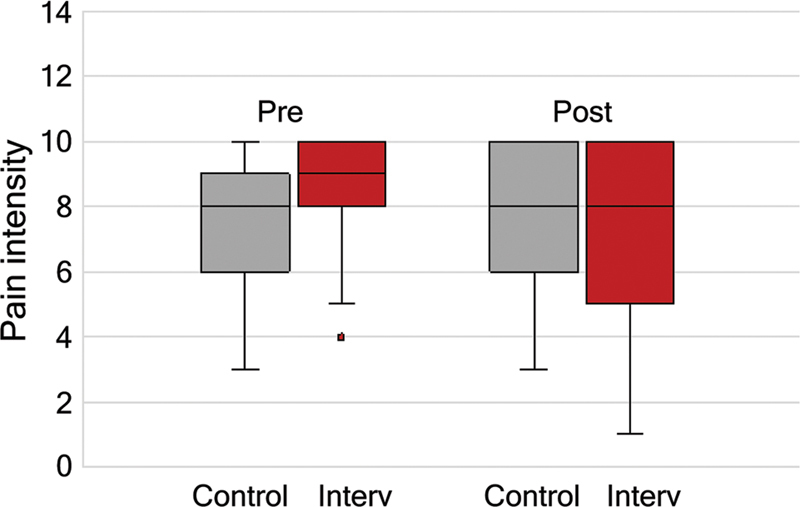
Pain intensity pre- and postintervention.

In the intervention group, most patients reported an improvement in pain frequency over the same period (55.9%), with a reduction in the frequencies “daily” (64.11%) and “4 to 6 times a week” (100%) and an increase in the frequencies “1 to 3 times a week” and “less than once a week” ( *p* < 0.001), showing a statistically significant difference between the pre- and postintervention periods. These findings are summarized in Tables 2 and 3 and Figure 1 .

In the control group, most patients maintained the same pain frequency between the pre- and postintervention periods (75%), with an increase in the frequencies “4 to 6 times a week” and “1 to 3 times a week,” although without statistically significant variation.

#### Pain intensity

In the control group, there was no statistically significant variation in headache intensity, while in the intervention group, it could be observed a reduction in the average intensity from 9 to 8 ( *p* < 0.001).

**Figure 2:**
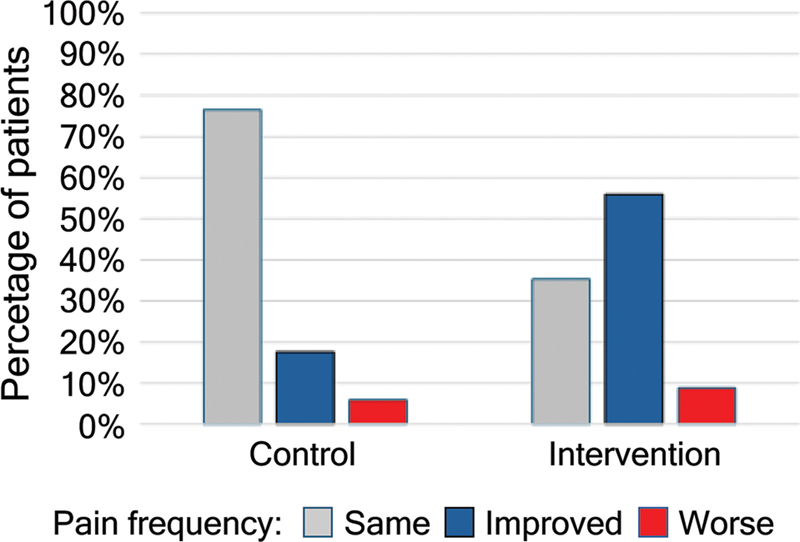
Comparison between pain frequencies in the pre- and postintervention periods between the control and intervention groups.

Statistically significant variations were observed between the control and intervention groups in two parameters: pain intensity preintervention, with an average intensity of 8.6 and a SD of 1.5 for the intervention group and with an average intensity of 7.7 and a SD of 1.9 for the control group ( *p* = 0.013); and reduction in pain intensity from pre- to postintervention, with an average of 0.1 and a SD of 1.4 in the reduction in intensity for the control group and with an average of 1.4 and a SD of 2.6 in the reduction in intensity for the intervention group. These data on pain intensity and variations between the pre- and postintervention periods are included in Table 1 and Figure 2 .

### Analysis of the scores of the standardized Epworth, Pittsburgh, and insomnia severity index questionnaires

From the results of these questionnaires, it was noticed that there was a statistically significant variation between the results before and after the guidance of the sleep psychoeducation measures in the PSQI ( *p* = 0.005) and ISI ( *p* < 0.001), while the ESS questionnaire did not present a statistically significant change ( *p* = 0.918), as shown in Table 4 .

**Table 4:** Results of the ESS, PSQI, and ISI questionnaires before and after sleep psychoeducation measures for the intervention group (n = 68)

Questionnaire	Assessment		Mean	SD	Median	Min.	Max.	*p* -value*
ESS	Pre		7.5	±5.9	6	0	22	
	Post		7.2	±5.9	6	0	24	0.918
	Reduction (pre–post)		0.2	±8.4	−1	−18	17	
PSQI	Pre		13.7	±3.7	14	5	20	
	Post		11.7	±4	12	4	20	0.005
	Reduction (pre–post)		2	±5.8	2	−14	15	
ISI	Pre		18.5	±5.4	19.5	6	28	
	Post		13.4	±5.8	14	1	28	<0.001
	Reduction (pre–post)		−5.1	±7	−5	−17	15	

Abbreviations: ESS, Epworth Sleepiness Scale; Max., Maximum; Min., Minimum; PSQI, Pittsburgh Sleep Quality Index; SD, standard deviation; ISI, Insomnia Severity Index.

Note: *Non-parametric Wilcoxon test, p < 0.05.

When analyzing the PSQI separately, according to each of its domains it becomes evident that the most affected aspects of sleep quality were subjective quality, duration, and disturbances ( Table 5 ).

**Table 5:** Results of the PSQI domains, comparison of pre- and postintervention scores in the intervention group (n = 68)

PSQI Domain	Assessment		Mean	Median	*p* -value*
**Subjective sleep quality**	Pre		2.15	0.76	
	Post		1.76	0.71	
	Reduction (post–pre)		−0.38	0.96	0.002
**Sleep latency**	Pre		2.38	0.91	
	Post		2.10	0.93	
	Reduction (post–pre)		−0.28	1.39	0.108
**Sleep duration**	Pre		2.16	1.03	
	Post		1.68	1.13	
	Reduction (post–pre)		−0.49	1.45	0.010
**Habitual sleep efficiency**	Pre		1.75	1.24	
	Post		1.32	1.20	
	Reduction (post–pre)		−0.43	1.85	0.046
**Sleep disturbances**	Pre		1.88	0.66	
	Post		1.62	0.71	
	Reduction (post–pre)		−0.26	0.97	0.030
**Use of sleep medication**	Pre		1.59	1.51	
	Post		1.62	1.50	
	Reduction (post–pre)		0.03	2.17	0.873
**Daytime disfunction**	Pre		1.75	0.97	
	Post		1.56	0.78	
	Reduction (post–pre)		−0.19	1.24	0.238
**PSQI**	Pre		13.66	3.70	
	Post		11.66	4.00	
	Reduction (post–pre)		2.00	5.80	0.005

Abbreviations: Max, maximum; Min, minimum; PSQI, Pittsburgh Sleep Quality Index; SD, standard deviation.

Note: *Nonparametric Wilcoxon test, *p* < 0.05.

## DISCUSSION

The findings suggest that sleep psychoeducation, delivered in a single session, effectively improved both chronic migraine symptoms (intensity and frequency) and sleep quality in patients with sleep complaints. The intervention, conducted in an outpatient setting by medical students, was practical and well-tolerated.

Previous studies have demonstrated that CBT-I significantly improves both insomnia and chronic migraine. 25 26 However, research on psychoeducation remains limited, though pilot studies have shown promising results. 27 28 This study aligns with prior findings, as intervention group patients reported a notable reduction in headache intensity and frequency, whereas over 70% of control group participants experienced no improvement. These results support the incorporation of psychoeducation into routine migraine care.

The relationship between sleep disorders and migraines is well-established, mainly because intensities and frequencies of migraine episodes are related to greater severity of insomnia; 14 however, the impact of brief sleep behavior interventions on chronic migraine requires further investigation. 27 28 Our results reinforce the role of behavioral sleep interventions as an adjunctive measure in chronic migraine management.

Statistical analysis using the PSQI and the ISI confirmed improvements in sleep quality and insomnia severity, and the intervention moved participants' mean ISI scores from a more to a less severe range of their standard clinical categories. 29 30 No significant changes were observed in daytime sleepiness ESS, which aligns with the study population's primary complaint of insomnia rather than hypersomnia. This clinical characteristic was confirmed by the screening test priorly conducted, in which none of the patients in the intervention group reported daytime sleepiness as a complaint.

Based on the results, it can be observed that the variation in PSQI scores within the intervention group between the pre- and postintervention periods, although statistically significant, corresponded to a modest reduction of only 2 points (from 13.7 to 11.7). However, when analyzed together with the Insomnia Severity Index, which decreased from 18.5 to 13.4, the findings indicate a clinically meaningful improvement in patients' sleep quality. Considering the previous scientific literature suggesting a bidirectional causal relationship between migraine and insomnia, it is likely that the observed changes in clinical headache characteristics were, at least partially, influenced by improvements in sleep quality. 9

The domains of the PSQI which showed the greatest variation (subjective sleep quality, duration, and disturbances) suggests that participants experienced not only a perceived decline in their overall restfulness but also measurable changes in both quantity and continuity of sleep. Such findings highlight the multifaceted nature of sleep alterations, emphasizing that both subjective perception and objective patterns are essential for understanding sleep health comprehensively. These variations across the three domains may reflect the effectiveness of the sleep psychoeducation implemented for participants in the intervention group.

### Practical implications and relevance

Sleep psychoeducation offers a viable and low-cost alternative that can be administered by nonspecialists, overcoming barriers to accessing CBT-I, which requires trained professionals and multiple sessions.

The epidemiological profile of our sample is consistent with migraine prevalence trends, which show higher incidence in women and middle-aged adults. 31 32 The predominance of female participants (88%) and the average age (51.8–54.5 years) likely reflect the chronic nature and delayed diagnosis of migraines in this patient population. 33

### Pharmacological considerations and limitations

No significant association was found between pharmacological treatments and migraine outcomes. Notably, in the intervention group, there was a reduction in botulinum toxin administration, possibly reflecting symptom improvement. Conversely, the control group required more anesthetic blockades postintervention, suggesting persistent symptom burden.

The present study's limitations include sample size constraints, potential confounding factors (concurrent medication adjustments, patient adherence to the recommended measures, and control group consisting of patients without sleep complaints), no-randomized design, short follow-up period, and absence of polysomnography to objectively define sleep disorders. However, pharmacological data were systematically recorded to minimize bias. Another limitation of the study was the absence of headache diaries for patients of both groups, which could have improved the objectivity of clinical variables' analysis.

When considering the potential contribution of placebo, the statistically significant improvements observed in the intervention group between pre- and postintervention assessments were substantially greater than those in the control group, reinforcing the likelihood of a therapeutic effect of the intervention.

A key strength of this study is that sleep psychoeducation was effectively conducted by medical students and may be an option in the management of patients with chronic migraine and insomnia. Future research should further investigate its role in chronic migraine management in patients with sleep complaints.

In conclusion, sleep psychoeducation, applied in an outpatient headache clinic, effectively improved chronic migraine symptoms (reducing pain intensity and frequency) and enhanced sleep quality. The intervention was easy to implement, reinforcing its practicality as an adjunctive nonpharmacological approach for managing migraines and sleep disturbances. It can be feasibly delivered during routine outpatient consultations. Incorporating sleep guidance into standard care may enhance symptom management and improve patients' overall quality of life.
